# Drug-development, dose-selection, rational combinations from bench-to-bedside: are there any lessons worth revisiting?

**DOI:** 10.18632/oncotarget.27931

**Published:** 2021-05-25

**Authors:** Alexander J. Bridges, Ranjit K. Mehta, Sushmita Shukla, Matthew J. Schipper, Theodore S. Lawrence, Mukesh K. Nyati

**Keywords:** dose selection, MTD, MED, drug development, AT13387

## Overview

One of the most intractable problems in drug development is getting an impartial and accurate evaluation of the agent one is working on, especially since patients are involved in clinical trials. It is particularly acute in the oncology field, where both cause and appropriate treatment of a tumor may partially be rational guesswork. In this situation, patients have a high likelihood of a sub-optimal outcome, notably when they, as profoundly impaired "salvage patients", end up in early clinical trials, receiving modestly characterized agents, which can do great harm if not judiciously used. These factors argue for caution when evaluating an agent, and there are several rules of thumb designed to terminate weak drug candidates as rapidly as possible. This precautionary stance is justified by the high failure rates of drugs in clinical trials. In this review, we argue that certain rules of the drug developmental paradigm are frequently misapplied, resulting in many good agents being discarded, and we provide an example of Hsp90 inhibitors. These “rules” are worth revisiting so that new cancer drugs can be developed and tested in a way that increases the overall success rates in patients.

## Challenges in cancer drug development

The aim of cancer drug development is to kill malignant cells while minimizing the adverse effects on normal cells. But, as the processes and molecules being targeted express in both types of cells to a different extent, this aim is always a balancing act, with extremely few “clean shots” which are not also targets in the patient's normal tissues. The development of a new treatment modality, especially a novel cancer drug, is arduous work and takes years from conception to apparent success (or failure). The process for new drug approval is prolonged, taking 5–15 years with billions of dollars of investment. It is a roller coaster with high attrition, mostly due to clinical efficacy, safety, or commercial concerns. Even when successful, later treatment failure due to the development of eventual drug resistance remains a major challenge, and the majority of patients with inoperable solid tumors still die from them. Bringing a new drug to the market is governed by stringent regulations. Projects can fail at any step of the drug discovery process, particularly during late-stage clinical trials. Despite outsiders' rather glamorous views of drug development, less than 10% of projects make it from Phase I to approval.

## First-in-class drugs versus me-too drugs

Developing a "me-too" drug, also known as a "follow-on" drug, when there are successful drugs already in the clinic, or preferably on the market, suggests that some of the science around the drug is well accepted. However, that does not mean it is well understood or even necessarily mostly correct. A "me-too" drug is not only attractive because the initial leads often come from somebody else’s patent application, but because the predecessors also provide a benchmark to measure the success of the project. Being "first-in-class" can have a considerable commercial advantage, but coming in later with a genuine "best-in-class" is usually more profitable. Also, if the first-in-class was not particularly good, there is not only a roadmap to develop the compound, but also benchmarks, which will almost guarantee regulatory approval if they are exceeded.

Novel drugs are potentially more valuable to society compared to “me-too” drugs, as they can target heretofore unmet medical needs. However, if one is developing a drug for a novel disease or a novel mode of action, the process is more challenging because one has to deal with much less settled science, and no guarantee that the new mechanism will work in humans in a way consistent with the animal studies. To find this out, extended testing of compounds is required, which leads to higher financial risks as more time and money are spent on novel drugs which do not make it to the market.

There are two ways in which one can deal with problems of this nature. First, follow a series of established rules and protocols algorithmically, and second, rely on an experienced researcher’s judgment or instinct. Most pharmaceuticals companies tend to emphasize the first. A series of rules and protocols are set up, and the project is continually sieved through them, looking for the failure of a particular screen to winnow out the losers. The more is known about a disease and a specific mode of action for treating it, the better this process should work, as the various initially guessed case-specific parameters should be improvable using internal or literature data. The second method is to rely on the judgment of a highly experienced research division leader. This method is much more practiced than admitted to, but history is not particularly kind to individual judgments. Both these methods do not necessarily bring the best available understanding and analysis of the problem when making decisions, especially regarding what should be advanced in the clinic and how it should be evaluated in patients.

In this review, we examine the application of few aspects of drug development that might explain why every single agent targeting Hsp90 failed in the clinic. These claims were evident in a recent publication from Mehta *et al*. [1]. For example, one expectation is that any new agent must show single-agent efficacy in the clinic before moving into advanced studies. This expectation is strengthened by regulators, as the most favored way to advance an anti-cancer drug to approval is to add it to the best supportive care (i.e., no actual pharmacological attack on cancer) and show single-agent activity in this setting. To meet this goal, the focus almost invariably shifts to using the maximum tolerated dose (MTD) to ensure the efficacy is not compromised. The MTD dose is the highest dose administered without significant adverse consequences of the test agent. It is also the dose generally given to patients in Phase 2 and 3 clinical trials and the clinic; therefore, it is also referred to as the Recommended Phase 2 Dose ("RP2D"). Early single-agent clinical trials are usually conducted in patients who are left with no other treatment choices, with the most resistant and recalcitrant tumors, and the success of therapy critically depends on an efficacy signal in this exceptionally difficult to treat population. Thus, the recommended drug dose is either the MTD or very close to it, to achieve the maximum therapeutic effect. However, MTD doses regularly lead to various adverse effects, and most oncology drug labels include recommended dose de-escalations for patients who find the therapy intolerable. It has been shown that lower doses of some metastatic breast cancer (MBC) therapies may be as effective as the MTD with less severe side effects [2]. Also, the lower doses usually allow patients to remain on treatment for an extended period due to lower toxicities. Therefore, it is crucial to balance safety and efficacy during dose selection in cancer treatment.

## Maximally tolerated dose regimen versus optimal dose regimen

It is crucial to consider the balance between treatment’s benefit and adverse effects in oncology drug research. In the current drug development process, the balance appears to have tilted towards toxicity, sometimes leading to the treatment being almost as damaging as the disease. Often, drug trials focus entirely on getting the FDA’s approval, rather than addressing the real benefit to patients. There is a need for change in this approach; however, identifying the optimum dosage or schedule is a complex process, and it involves time and a trial-and-error approach [3]. Unfortunately, commercial interests primarily drive the current drug development process. Very few Phase 2 trials examine the Minimally Efficacious Dose (MED), and most drug approvals are based on trials conducted using the MTD, which later gets incorporated into the patient treatment. Pharmaceutical companies are reluctant to measure low dosing potential since the cost of goods is immaterial, and the use of submaximal dosing raises the spectre of failure due to lack of efficacy. Thus, such trials would have to be larger, longer, and costlier, delaying regulatory approval, and, in the case of a drug with a novel mechanism of action, risking the loss of first to market advantages. Furthermore, classical oncology MTD determinations in Phase 1 trials, which often involve 3+3 designs and less than 20 patients, are statistically more likely to get the MTD wrong. Lastly, if one can get approval without extra trials, which in turn might uncover new problems, the commercial logic argues not to do said trials, and outside entities are not interested in funding such studies either. Therefore, there is a need to "re-engineer" the drug regulatory system and focus on patients' betterment rather than seeking just approval for new drugs. Different early phase study designs have been proposed to improve this situation [4, 5].

In addition to redirecting the research to put less emphasis on MTD and more on MED, cancer drug development also needs to focus on combination therapy. Combination therapy requires the optimum use of drugs that can enhance a patient’s overall wellbeing by targeting different paths on which cancer cells survive. For a successful combination, deeper understanding of the effects of individual therapeutics is a must to produce selective "synthetic lethality". If the host organism (patient) has already received enough of one component to bring it to near-fatal toxicity, there is very little wriggle room for any adverse effects of a second agent, especially if it is also dosed at or near to an MTD. It is in the area of combinations that MED information is most important, and since the belief in magic bullets has largely evaporated, it is self-evident that optimal treatments will almost always be combinatorial.

## Single-agent versus combination therapy

Anti-cancer drugs can be given as monotherapy or combined with other agents to achieve a better therapeutic index. Combination therapy is expected to be more effective than monotherapy. Sometimes the most promising single agents can fail because tumor cells can acquire resistance by activating alternative pathways. That is precisely why the combinations of different modalities are the only way to move forward for many resistant cancers. However, when MTD doses are combined with other agents, it is not hard to believe that often such combination results in a significant and immediate increase in off-target effects and toxicity. It is a fact that designing combination strategies is a complex process where the emphasis is given to reduce overlapping toxicities due to different mechanisms of action of the drugs and differences in pharmacokinetic profiles.

Most combination trials are designed to modify only the dose of a single component treatment with doses for the other treatments fixed at or near to their single-agent MTDs. While this is partly understandable due to the larger sample sizes required to perform two-dimensional dose finding, it also represents a missed opportunity to estimate an optimal pair (or triplet) of dose levels for each drug that maximizes treatment efficacy while maintaining dose limiting toxicity (DLT) to acceptable levels. Of course, this means that if there are any overlapping toxicities, patients will experience enhanced side effects of treatment, which may compromise any potential benefit. In addition, the choice of drugs in combination therapy is also influenced by factors beyond scientific rationale. For example, sometimes a pharmaceutical company designs a trial to gain an extension of patent protection on its agents, which a method of use patent could provide, or sometimes two marginally active agents are combined from business partners in the hope that two therapeutic wrongs will make a commercial right. Also, the partner could be off-patent from elsewhere, in which case a combination patent could bring in revenues, especially if one could reformulate the mixture. Of course, with such thinking behind many combination trials, the failure rate is exceptionally high, reinforcing the original "must-have single-agent activity" mantra.

As discussed in the featured example below, even the most pleiotropic drugs are expected to induce certain biochemical effects at lower doses than other effects, and frequently well below any efficacy dose. Such effects should be paid great attention in designing any combination therapy because they are inescapable consequences of using the drug at all meaningful doses. These low dose effects need to be considered both in tumor cells, where they will underpin a facet of the desired efficacy, and in normal tissues, where they could provide biomarkers of potential side effects. Proper consideration of these effects for each of the agents in a potential combination can offer insights to determine the ideal efficacy regimen as well as determine doses to minimize DLTs. Failure to do both parts of this analysis, what benefit will the combination provide for anti-tumoral efficacy, and what effect will both agents have in inducing unwanted toxicity? Perhaps, this is why so many combination trials have been run combining two similar action agents at close to their MTDs. For example, the above-discussed idea can explain why patients experienced greater toxicities with minimal benefits when afatinib and cetuximab at their MTD were combined to treat patients with stage IV or recurrent EGFR-mutant NSCLC. This combination did not improve clinical outcomes compared to afatinib alone but resulted in greater toxicity which led to frequent dose reduction and treatment discontinuation [6]. Such findings shed light on the need to develop optimal doses of drugs when used in combination to improve the quality of life while retaining the efficacies, which can only be achieved by understanding which actions of the drug are useful to combine, and what the lower threshold of utility will be for each activity brought into the combination.

## Hsp90 inhibitors

Heat shock protein 90 (Hsp90) stabilizes many oncogenic mutant proteins, and inhibition of Hsp90 has been investigated as an anticancer target for several decades. However, no Hsp90 inhibitors have been FDA approved to date due to their limited efficacy. Geldanamycin was first identified in 1970 [7], and its anti-tumor activities were first reported in 1977 [8]. In 1986 it was shown to block activation of c-Src in transformed mammalian cells, and for several years, it was reported on as an inhibitor of tyrosine kinases (TKs). Only in 1994 its target was identified [9]. Similarly, ansamycin antibiotics herbimycin (A&B) were identified in 1980, and their anti-tumor effects were reported in 1984. It was shown to block v-Src in 1985 and was reported on very extensively as a “specific inhibitor” of multiple different TKs, and well over 200 papers were published on this before it was identified as an Hsp90 inhibitor, along with geldanamycin [9, 10]. The first Hsp90 inhibitor to enter the clinic was 17-allylamino-1-deoxy-geldanamycin (17-AAG), which entered Phase 1 in 1999, and had at least 31 clinical trials, including at least 13 combination trials before being abandoned [11]. Eighteen Hsp90 inhibitors had entered the clinic by 2014, and by 2018, only 5 were still in development. Currently, in https://clinicaltrials.gov/ only PU-H71 and XL888 of this cohort may still be in clinical trials (one entry each), and three new inhibitors appear to be in the clinic, albeit one for psoriasis, so presumably at a very sub-efficacious cancer dose. Thus, overall, the development of Hsp90 inhibitors to date can only be described as a costly failure.

The list of proteins that Hsp90 interacts with is vast. It includes at least 40 other chaperone proteins, around 100 transcription factors, over 200 kinases, and over 450 other proteins, from the hERG channel to DNA polymerases [12]. If Hsp90 is required for all of these proteins to fold and locate successfully, the extraordinary cytotoxicity of Hsp90 inhibitors would not be surprising, and if only a quarter of those proteins have an absolute requirement for Hsp90, high cytotoxicity would still be expected in normal cells as well as transformed cells. If we prevent Hsp90 from binding and subsequently hydrolyzing ATP, we reduce the concentration of functional Hsp90, but without reducing the overall amount of Hsp90 protein in the cells. Different proteins rather self-evidently use different surfaces to bind to any given Hsp90 binding site, and as the interaction surfaces differ, so must their absolute affinity for Hsp90. There is usually a large amount of Hsp90 in cells, often more than any individual proteins that Hsp90 is chaperoning, but greatly less than the total number of all the proteins that can bind with Hsp90. This suggests that there will always be competition for available Hsp90 between its various substrates, and on average, those with higher binding affinity will occupy more Hsp90 than those with lower binding affinity. If inhibitors do not allow Hsp90 to form productive conformations to bind client proteins, the effect will be to reduce the effective concentration of Hsp90, which will mean that on average poorer binding clients will be prevented from being chaperoned before the better binding clients. However, if an Hsp90 inhibitor traps Hsp90 in a form where it can bind clients in the normal way but cannot fold or chaperone them to their appropriate cellular location, the Hsp90 may act as a dominant-negative, and the main "victims" of the inhibitor could be high-affinity clients, which are now bound in an unproductive Hsp90 complex. One can hypothesize several further wrinkles on this theme, for example, with different inhibitors possibly inducing different conformations, and the end results become very difficult to predict or extrapolate from historical data sets.

Regardless of mechanistic speculation, one can examine the problem experimentally by taking tumor cell lines of interest and treating them with considerably lower than cytotoxic doses of Hsp90 inhibitors, and then analyzing for the proteins which are significantly attenuated at the lowest biochemically active dose. Such data can define the requirement of a certain biological effect(s) associated with the efficacy. These effects can be enhanced by careful dose-escalation while avoiding the toxic sequelae. The former comports to square pegs for square holes and will almost certainly be the easiest way to go. The choice of an agent to produce additive toxicity in tumor cells, but not in the normal cells will, of course, depend upon what the low dose effects are, and it is certainly possible that one will end up with sets of activities that do not appear to be useful to try to combine. In which case, on to the next project.

## Combination study with AT13387 and radiation

Mehta, et al. used an Hsp90 inhibitor AT13387 (onalespib), which has shown exciting activity in clinical trials, but even at the MTD does not have an approvable efficacy profile [1]. AT13387 has certain interesting properties that make it relatively non-toxic at a therapeutic dose. It can be dosed only once a week, and in xenograft models, there is strong tumor retention, such that the level in tumors are at least 10–fold higher than the levels in plasma. The MTD was 120 mg/m2 on the biw schedule and 260 mg/m2 on the qw schedule, with a 3 week on/1 week off cycle. In mice, the efficacy dose was 90 mg/kg qw, and lower doses were not effective.

A 3 μM cellular dose has been studied quite extensively and is cytotoxic in many tumor cells, including the HNSCC cell line, UMSCC74B. By looking at a proteomics scan in this cell line, the authors discovered that over 35% of the examined proteome was significantly perturbed at this dose. Notably, Hsp70, the standard biomarker for Hsp90 inhibition, was upregulated over 10-fold. The authors then looked at Hsp70 levels at a sub-therapeutic concentration of AT13387 and determined that 100 nM was the lowest dose which induced a doubling of Hsp70 levels, and only 33 proteins were significantly downregulated at that concentration, at least a third of which are involved in DNA damage repair. This suggested that DNA damaging agents might cause prolonged damage in the presence of low levels of AT13387. This was examined by analyzing the dose curves of radiation in the presence and absence of 100 nM AT13387. In 11 cell lines, the combination showed good enhancement ratios, leading to robust cell killing. DNA damage, measured by counting γH2Ax foci, only showed a modest immediate increase. However, DNA damage repair was considerably slowed in the AT13387-treated cells, leading to overall significant increases in DNA damage exposure, and consequential cell death. Three tumor xenografts, UMSCC74B, SCCVII, and MiaPaCa-2, were examined *in vivo*, treating with AT13387 at about half the single-agent efficacy exposure, or radiation (2Gy 5x/week) or a combination, whereby the same dose of AT13387 was used, along with a 60% reduced radiation dose. In UMSCC74B and SCCVII tumors, neither single agent had useful activity, but the combinations were effective with tumoristasis during dosing and for 1–2 weeks post-dosing. Lastly, in the MiaPaCa-2 tumors, AT13387 was inefficacious, but radiation retained tumoristasis out to about 20 days after dosing, and then regrowth occurred, parallel to control. The combination saw modest regressions, and regrowth was more delayed than for higher dose radiation alone, and less rapid when it eventually occurred. Thus, in all three tumors, the combination was highly effective, with well below MTDs being used for both the agents.

Subsequent to this report, another publication has drawn similar conclusions for the combination of AT13387 with cisplatin, another DNA-damaging agent [13]. The therapeutic potential of utilizing Hsp90 inhibitors most effectively yet remains to be realized. The best use of Hsp90 inhibitors may not be using them at high doses to kill cancer cells but using them at low doses in combination with other targeted therapies, which exploit their low dose (subtherapeutic) biochemical profiles.

## CONCLUSIONS

Tremendous amounts of work go into the development of new drugs, and especially new classes of drugs. A drug should have a potential niche to fill in both the medical armamentarium and the marketplace. We would like to have highly efficacious, clean agents to cure all our ills. In many therapeutic areas, we have excellent single agents, which means that it is tough to bring in a competitor, let alone a combination of agents, to most patients in that particular therapeutic domain. For example, hypercholesterolemia is a solved pharmaceutical problem for the majority of patients. It is reasonable to ask what uncured medical condition any potential new treatment addresses and, if it does not, how it can compete with generic statins. Therefore, in a case like this, stringent scrutiny is unlikely to miss anything significant. To avoid too many futile exercises, we have many rules of thumb that are useful to consult and ponder, but when raised to the level of holy writ and applied inappropriately where background circumstances are very different, we can often do more harm than good.

In oncology, where the unmet need is still large, new ways of testing potential therapies must be considered. This is especially true for solid tumors, in which if the tumor is not contained locally, and becomes metastatic at some point before or during treatment, most patients will die of the disease. Overall, we probably know more about the mechanisms that drive cancers than almost any other disease. We understand that most solid tumors are not genetically homogenous. So, we should expect that many of them will not respond particularly well to single agents, and conversely not be surprised when even in cases where dramatic regressions are seen, the tumors overcome the successful agents and come back in 6–18 months. So, we should be thinking of our novel agents from the beginning as a tool that can be used in combination with other agents to increase the combination’s overall therapeutic power, but at doses where such efforts will not be stymied by already well-documented DLTs. Such felicitous outcomes will not be found either by chance or commercial need but by a thorough understanding of the basic science involved.

Mehta *et al*. represent an excellent example of the type of study that may benefit patients and allow drug developers to get the maximal return from the enormous efforts and money sunk into developing targeted oncology agents.
